# Livelihood Diversification and Residents’ Welfare: Evidence from Maasai Mara National Reserve

**DOI:** 10.3390/ijerph20053859

**Published:** 2023-02-21

**Authors:** Qi Sun, Chao Fu, Yunli Bai, Ayub M. O. Oduor, Baodong Cheng

**Affiliations:** 1 School of Economics and Management, Beijing Forestry University, Beijing 100083, China; 2 Key Laboratory of Ecosystem Network Observation and Modeling, Institute of Geographic Sciences and Natural Resources Research, Chinese Academy of Sciences, Beijing 100101, China; 3 United Nations Environment Programme-International Ecosystem Management Partnership (UNEP-IEMP), Beijing 100101, China; 4 Department of Applied Biology, Technical University of Kenya, Nairobi P.O. Box 52428-00200, Kenya

**Keywords:** livelihood diversification, strategies, income, Maasai Mara National Reserve, off-farm activities

## Abstract

The contradiction between environmental protection and livelihood development is becoming increasingly serious for most protected areas in developing countries. Livelihood diversification is an efficient way to increase household income to alleviate poverty related to environmental protection. However, its impacts on household welfare in protected areas have rarely been quantitatively explored. This article investigates the determinants of four livelihood strategies in the Maasai Mara National Reserve and explores the association between livelihood diversification and household income and its heterogeneities. Based on the sustainable livelihoods framework and the information collected from 409 households through face-to-face interviews, this study adopted multivariate regression models to obtain consistent results. Results show that the determinants of the four strategies differed. Natural capital, physical capital, and financial capital had significant associations with the probability of adopting the strategy of livestock breeding. Physical capital, financial capital, human capital, and social capital were associated with the probability of adopting the joint strategy of livestock breeding and crop planting and the joint strategy of livestock breeding and off-farm activities. The probability of adopting the joint strategy of livestock breeding, crop planting, and off-farm activities was associated with all five kinds of livelihood capital except for financial capital. Diversification strategies, especially those involving off-farm activities, played greater roles in raising household income. The findings indicate that the government and management authority of Maasai Mara National Reserve should provide the households around the protected area with more off-farm employment opportunities to increase the welfare of local residents as well as to utilize natural resources appropriately, especially for those located far away from the protected area.

## 1. Introduction

Considering the global biodiversity crisis, there is an urgent call to protect the world’s oceans and land by 2030 [1]. Particularly in developing countries, the establishment of protected areas (PA) is being adopted as the most feasible strategy for decreasing undesirable effects on biodiversity due to external pressures [2,3,4,5]. In terms of geographical characteristics, PAs are usually distributed in relatively poor and remote areas, where the local residents are more dependent on natural resources [6,7,8]. Due to the various rigorous restrictions of PAs, residents in surrounding communities may conduct illegal logging and participate in the overexploitation of resources to sustain their livelihoods, which further aggravates the problem [9,10]. As a result, the contradiction between protection and livelihood development is becoming increasingly serious, which is one of the main drivers of protection failure [11,12]. 

Therefore, the impact of the PAs on the livelihood of local residents has been the focus of attention in recent years. Most studies find that the distance to the PA is closely related to the welfare of residents. More specifically, compared to households living a considerable distance from the PAs, adjacent households are poorer and perceive widespread negative effects, because they have worse access to markets and social services and fewer employment opportunities [13,14,15,16,17]. However, some scholars put forward that households living closer to PAs may receive some benefits. For example, distance to the boundary of a PA is negatively associated with environmental income, implying that households living closer to the PA have a higher environmental income due to benefits offered by ecotourism [18,19,20,21]. 

Many authors have explored methods of balancing environmental protection and poverty alleviation based on the theoretical foundation of the sustainable livelihoods framework [22]. Following this framework, households use a range of assets to achieve positive livelihood outcomes, in which different livelihood strategies are adopted to combine assets for survival. In general, households can utilize different strategies to manage risk and cope with shocks. Specifically, livestock breeding, crop planting, and off-farm activities (e.g., tourism) are the most common strategies for residents around PAs in Africa [23,24,25,26,27,28]. Furthermore, the process by which rural households construct a diverse portfolio of activities and assets in order to survive has been defined as “livelihood diversification” [29]. The choice of diversification, both on- and off-farm, can be considered a safety-net, providing livelihood options in the context of extreme events, especially for the poor [30]. In other words, the diversification of livelihood strategies can significantly improve household income, notably so for low-income smallholders, and contribute to reducing inequality [31]. In particular, diversification, including off-farm work, was confirmed as a welfare-enhancing strategy for most communities around PAs in developing countries [31,32,33,34,35].

The Maasai Mara National Reserve (MMNR), as the most famous wildlife reserve and highest-earning wildlife area in Kenya [36], has been widely studied. There is a common research consensus that the conflict between man and nature is still serious in the MMNR [37,38,39]. Tourism brings some benefits to local communities. However, there are many cases where local households feel disgruntled towards conservation efforts, especially when they are faced with a series of restrictions, such as being prohibited from cultural practices or using natural resources, e.g., for grazing [24,26]. As a result, the best way to balance the relationship between protection and development is an important issue for MMNR.

When examining the most relative studies on the effect of livelihood strategies on livelihood outcomes, there are still certain research gaps worth narrowing. Firstly, recent studies have mainly examined the relationship between livelihood strategy and livelihood outcome. Studies that assess the motivation of livelihood strategy decisions are fairly rare. However, the research based on behavioral motivation is more conducive to supporting evidence-based policies. Secondly, although many studies have studied residents’ welfare around MMNR, few have concentrated on the impact of specific diversification strategies on livelihood outcomes, especially diversification involving off-farm activities. Thirdly, the mechanism underlying the effect of diversification on household income around MMNR is yet to be explored, which is meaningful for implementing more targeted policies.

This paper contributes to the growing research on livelihood diversification and welfare around PAs in developing countries. The relevance of this study is twofold: First, the determinants of different livelihood strategies around MMNR are assessed, which is necessary to expand on studies associated with households’ behavioral motivation around PAs in developing countries. Second, the mechanisms of diversification association with household income for residents living both adjacent to and further away from the PA are explored, which helps to provide targeted policy implications for poverty alleviation and community development around PAs. 

The rest of the paper is organized as follows. Section 2 provides the literature review and the theoretical framework, which guides the empirical analysis. Section 3 presents the methodology, including sampling and data collection, variables, and model specification. Section 4 shows the results of descriptive analysis. Section 5 explores the empirical results from the multivariate regression. Section 6 concludes and draws out the relevant policy implications of the analysis.

## 2. Literature Review and Theoretical Framework

### 2.1. Literature Review

The utilization of various capital influences a household’s choice of livelihood strategy [40]. Farming and off-farm activities are generally the most common livelihood strategies for households [41,42], while livestock breeding is popular in most African countries [23,27,43,44,45]. Many scholars have explored the roles of different livelihood strategies in improving the income of residents around PAs and have demonstrated mixed results. Maasai derive returns from livestock, non-farm work, and rain-fed cultivation, as well as from wildlife and from irrigated or upland farming, in some cases [32]. Specifically, income from the sales of livestock and livestock products remains the major source of livelihood in Maasai Mara [46]. However, other studies have put forward that actual income from livestock was small [47]. That being said, a survey indicated livestock production to be the primary source of subsistence for the Maasai, while those intent on taking up cultivation constituted more than half of the respondents [48]. 

As for off-farm activities, tourism activities have made a significant contribution to social welfare and have become an important source of revenue for some farming households located close to MMNR [38,47]. However, groups of farmers were choosing a higher and more consistent income from farming or leasing land out to large-scale contractors over limited and low returns from tourism with little prospect of the situation improving [49]. Furthermore, very little of the tourism income reaches the communities surrounding PAs, and income from tourism-based activities only contributes to a small proportion of households [26,50]. The inconsistency of these conclusions may be related to the sampling method. For example, some studies adopted a snowball approach, while others used group interviews, which provided various results.

Livelihood diversification around Maasai has been explored widely. For instance, Thompson et al. found that Maasai pastoralists in Kenya were rapidly diversifying their livelihood activities [49]. Maasai derived their main livelihoods (and sometimes considerable income) from farming, wildlife tourism, and/or the leasing of land for large-scale cereal cultivation. Household incomes were supplemented by tourist campsite rents, “dividends” from local wildlife trusts, employment in lodges, revenues from “cultural manyattas”, cultivation leases, and remittances from family members now living in the cities, and this diversification has brought wealth into the communities for family economic improvement [47]. There was less diversification, as most families kept livestock and had few, if any, sources of off-farm income among the Maasai of Kenya and Tanzania [51]. Maasai continue to aspire to keep livestock, irrespective of livelihood diversification, and livelihood diversification was sometimes aimed at building or maintaining herds [52]. 

The qualitative research on the relationship between diversification and household welfare around MMNR found that diversification was positively correlated with household welfare [33,34,35,53]. In addition, off-farm diversification is of extreme importance for many Maasai households [32]. If a household head has prior experience with off-farm work, this has a positive effect on income in all diversification groups, that is, the off-farm occupation has a significant and positive impact on income in the high-diversification group [31]. The barriers and success factors related to effective crop and livestock enterprise diversification reaffirm the importance of diversification beyond the farm into off-farm income sources [35]. It is noteworthy that diversification may lead to a decline in family welfare in some other areas. For example, Asfaw et al. [54] investigated the empirical linkages between crop and livelihood diversification strategies, extreme weather events, and household welfare and found that the improvement of diversification level reduced professionalism, resulting in the decline of household income. Overall, the association between diversified livelihood strategies and livelihood outcomes around MMNR has not been quantitatively confirmed.

### 2.2. Theoretical Framework

“Livelihood” was originally defined by R. Chambers and G. Conway [55]. Subsequently, the Department for International Development (DFID) proposed a more complete theoretical framework, that is, the sustainable livelihoods framework (SLF), which presents the main factors that affect people’s livelihoods and establishes typical relationships between livelihood capital, activities, and outcomes [22]. The livelihood framework identifies five core asset categories or capitals, including human capital, natural capital, financial capital, physical capital, and social capital. Human capital is usually indicated by the number of laborers and their education level as well as health condition, factors which are closely related to household members’ ages [10,14]. Natural capital is generally measured by the area of land owned or operated by the household, mainly referring to arable land [56,57]. Financial capital is usually indicated by the access to inclusive finance [11]. Physical capital is measured by the access to equipment and infrastructure, such as vehicles and agricultural production tools [40]. Social capital is generally indicated by membership of organizations or parties as well as social connections [10,57]. Households use this set of assets to obtain positive livelihood outcomes. Further, they have to find innovative ways of developing and combining what assets they have (i.e., livelihood strategies) to ensure survival [22,55]. 

As for livelihood strategies, the determinants of different strategies adopted by households are different. More specifically, the strategy of livestock breeding depends more on natural capital due to the demand for grazing areas, while households with less natural capital are more inclined to engage in off-farm activities [58,59]. As to livestock breeding and crop planting, two primary agricultural activities, the former depends more on financial capital since it needs a relatively large investment. The agricultural production tools related to livestock breeding are simpler than crop planting, so the impact of physical capital may be smaller for the former. Households with more vehicles have the convenience of being able to go out and are more likely to engage in off-farm activities, which in contrast, may reduce agricultural activities, such as livestock breeding. Moreover, households with high financial capital tend to invest more money in off-farm activities, since they require much capital and have higher returns than agricultural activities. The strategy involving off-farm activities is highly dependent on human capital, especially education level [60,61,62]. In addition, the impact of social capital on off-farm strategies is also bigger than that of agricultural strategies [54]. Therefore, the first hypothesis of this study is proposed as follows.

**Hypothesis** **1:** *Different livelihood strategies have different levels of dependence on livelihood capital.*

More specifically, the major livelihood strategies of residents around PAs and their dependence on livelihood capital are subdivided in accordance with four sub-hypotheses.

**Hypothesis** **1a:** *The strategy of livestock breeding relies on physical capital, natural capital, and financial capital.*

**Hypothesis** **1b:** *The joint strategy of livestock breeding and crop planting relies on human capital, social capital, physical capital, and financial capital.*

**Hypothesis** **1c:** *The joint strategy of livestock breeding and off-farm activities relies on human capital, social capital, physical capital, and financial capital.*

**Hypothesis** **1d:** *The joint strategy of livestock breeding, crop planting, and off-farm activities relies on human capital, social capital, physical capital, and natural capital.*

According to SLF, household income is a direct reflection of livelihood outcomes. Different strategic choices have different impacts on household income. It is common knowledge that household income from the non-agricultural sector is often higher than that from the agricultural sector. Moreover, since agriculture is vulnerable to various natural disasters, the risk of agricultural activities is higher; especially as agricultural income may be lower than that from off-farm activities [30,31,32,33]. Even regarding agricultural activities, the strategies of livestock breeding and crop planting also differ in the impact on household income. Livestock breeding is a capital-intensive activity with higher returns than crop planting. Meanwhile, crop planting is more likely to be affected by natural disasters compared to livestock breeding, resulting in household income decline. Furthermore, in Africa, the disadvantages of crop planting, such as the low quality of cultivated land, are more obvious than those of livestock breeding. Hence, two other hypotheses of this paper are proposed.

**Hypothesis** **2:** *The joint strategy of livestock breeding and off-farm activities as well as the joint strategy of livestock breeding, crop planting, and off-farm activities has a bigger impact on household income than the strategy of livestock breeding alone.*

**Hypothesis** **3:** *The joint strategy of livestock breeding and off-farm activities has a greater impact on household income than the joint strategy of livestock breeding, crop planting, and off-farm activities.*

**Figure 1:**
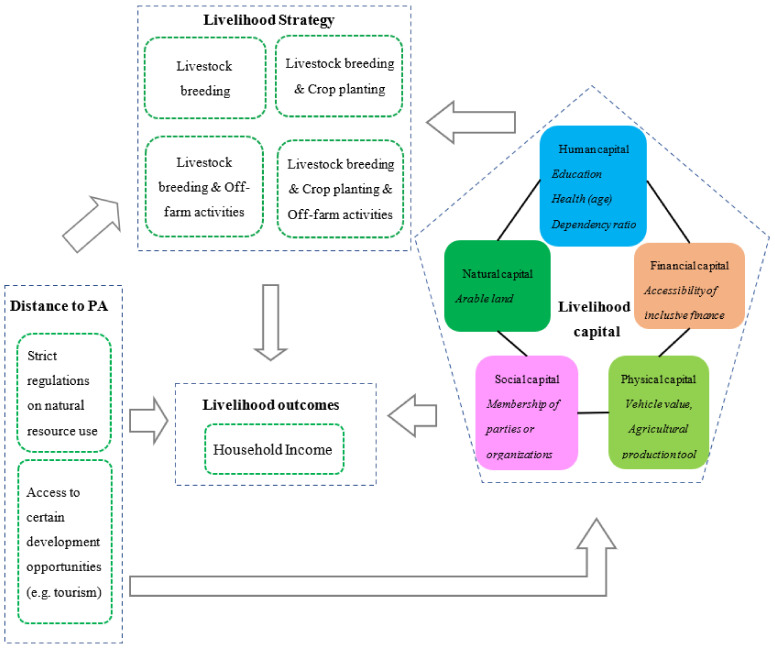
Conceptual framework of this study.

Based on the above analysis, the following conceptual framework of this study was developed (Figure 1). The government and management authority of Maasai Mara National Reserve (Kenya Wildlife Service, Nairobi, Kenya) are aiming to reduce the resource dependency around the PA by improving the income level of surrounding households through strict regulations and more access to certain development opportunities (e.g., eco-tourism). Such interventions affect livelihood capital, which can influence livelihood strategies and result in a change in income level for households around the PA. Furthermore, the impact of different strategies on household income may differ between households living adjacent to and distant from the PA in terms of management requirements. The complete logical analysis is shown in the conceptual framework of this study (Figure 1). An empirical model was built based on this framework; see the next section for details.

## 3. Materials and Methods

### 3.1. Sampling and Data Collection

The study uses the primary data collected by the authors in January–March 2017 at the sites around MMNR, which is located in the border region between southwest Kenya and Tanzania and is connected with the Serengeti National Park [63]. The reserve covers an area of 6400 square kilometers and is composed of open grasslands, woodlands, and riverine forests, accounting for about 25% of the Maasai Mara ecosystem. The remaining 75% of the ecosystem is unprotected land [26,63]. The MMNR is a major tourist destination in East Africa, and some of the main tourist activities include wildlife viewing and photography, camping, and cultural tourism [64]. There are strict restrictions on activities in the reserve, such as forbidding human habitation, grazing, and the logging and utilization of natural resources. The rural poverty level of the Maasai Mara ecosystem is very high, so it is necessary to analyze the livelihood strategy choices that affect the household welfare of communities surrounding the reserve.

**Figure 2:**
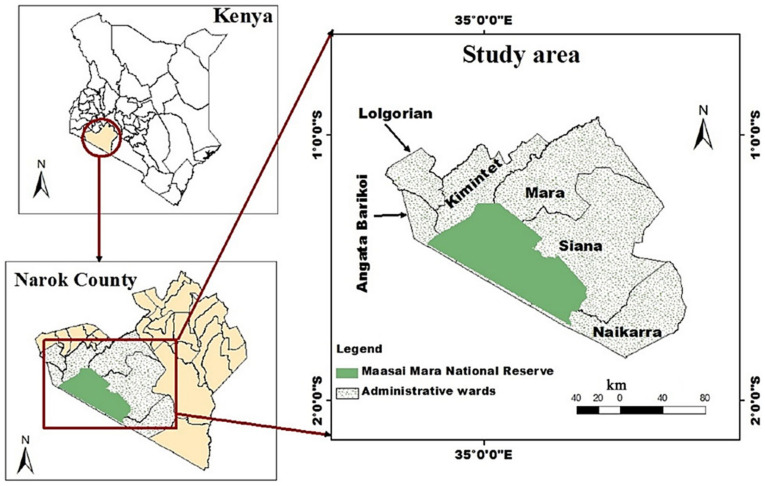
Study sites around MMNR. Note: this figure is cited from Mojo et al. [23].

We conducted the questionnaire surveys on 423 households in six wards (administrative districts, including Angata, Kimintet, Lolgorian, Mara, Naikarra, and Siana) through face-to-face interviews (Figure 2). The households were selected using the stratified random sampling method. Firstly, according to the proportions of impoverished populations released by the Kenya National Bureau of Statistics [65], all six wards were divided into three groups. Secondly, we used the topographic map of the District Land and Survey Office to select all villages within 25 km from the boundary of the PA in each ward. The survey included 72 villages, and the number of households surveyed in each village ranged from four to eight, for small and large villages, respectively. According to the list of villagers obtained from the local administrators, households were randomly selected for interview and each household was assigned a unique code. To avoid response refusal, we randomly selected alternative households. See Mojo et al. [23] for detailed sampling. Due to missing data, the information for 409 households was ultimately used as the sample of this study. We used a 5 km radius as the boundary for households located adjacent to and at a distance from the PA, according to a previous study, which found that such a distance covered the localized impacts of a protected area [23].

Based on the conceptual framework of this study, the authors designed the questionnaire and trained the interviewers. The interview questionnaire consists of six modules, in which demographic information, livelihood activities, social activities, financial, and basic information of households were collected for this study. In the demographic information module, age, gender, and education level were collected. In the livelihood activities module, the information on off-farm employment for each laborer in the household, the farming activities and income (such as crop planting and livestock herding), and the types and income from family businesses were gathered. In the social activities module, the expenditure for socializing and membership in a community group were documented. Financial information, including savings in banks and loans, was gathered. The basic information of a household, including household address and its distance to the MMNR, was collected.

With a rich amount of information collected, we defined and measured the variables. Specifically, key variables included types of the livelihood strategies and per capita household incomes. Control variables included the age and education level of the household head, household dependency ratio, household contribution to social activities (membership fee to community groups such as church and chama), household socializing expenditure (entertainment of friends and family), household vehicle value, household agricultural production tool value, household farmland area, household savings and loans, and distance of the household to the PA boundary. Details are shown in Table 1.

### 3.2. Model Specification

This study investigates the determinants of different livelihood strategies and assesses the effects of livelihood diversification on household welfare proxied by total income around MMNR based on the data collected by face-to-face interviews from 409 households. First, based on the sustainable livelihoods framework, we used multinomial logistic regression (Mlogit) to analyze the determinants of four livelihood strategies and explore the relationship between different livelihood capital and strategies. Second, we examined the association between livelihood diversification and household income and its heterogeneities using the ordinary least squares (OLS) model. 

Firstly, Mlogit was conducted to examine the determinants of different livelihood strategies. In empirical research, the average marginal probability effect is usually used to measure the association between explanatory variables and the occurrence probability of a type of strategy, which, in this research, was then used to obtain the prediction probabilities of different livelihood strategies. The model was specified as follows:

(1) $$Strategy_{i}=\delta H_{i}+\eta S_{i}+\zeta P_{i}+\theta N_{i}+\tau F_{i}+\rho L_{i}+\gamma R_{i}+\varepsilon_{i}$$

In Equation (1), $Strategy_{i}$ indicates the types of livelihood strategies for the household i. According to the information on livelihood strategy we collected, there are four values of this variable. If the household only adopted the strategy of livestock breeding, it equals to 1. If the household adopted the joint strategy of livestock breeding and crop planting, it equals to 2. If the household adopted the joint strategy of livestock breeding and off-farm activities, it equals to 3. If the household adopted the joint strategy of livestock breeding, crop planting, and off-farm activities, it equals to 4.

H represents the vector of human capital variables, including the age and education level of household head and the household dependency ratio. S represents the vector of social capital variables, including the membership fee to community groups, such as church and chama, and socializing expenditure (entertainment of friends and family). P represents the vector of physical capital variables, including vehicle value and agricultural production tool value. N represents the vector of a natural capital variable, including farmland area. F represents the vector of financial capital variable, including whether the household has savings in banks or loans. L represents the distance from household address to the PA. R is a vector of five dummy variables of the ward where the household is located. δ, η, ζ, θ, τ, ρ and γ are the vectors of the coefficients used to measure the effects of explanation variables on livelihood strategy, respectively. $\varepsilon_{i}$ is the error term.

Secondly, a reduced form of income function was built using the OLS model to explore the association between livelihood diversification and household income around MMNR. The model was specified as follows:

(2) $$lnperincome_{i}=\alpha +\beta Strategy_{i}+\delta H_{i}+\eta S_{i}+\zeta P_{i}+\theta N_{i}+\tau F_{i}+\rho L_{i}+\gamma R_{i}+\varepsilon_{i}$$

In Equation (2), $Strategy_{i}$, H, S, P, N, F, L, and R are the same as those in Equation (1). β is the coefficient that captures the effect of livelihood strategies on household income. α is the constant term. The description of explanatory variables is shown in Table 1.

## 4. Results

### 4.1. Descriptive Results

There are four livelihood strategies among the households in the sample as above-mentioned and most households adopted joint livelihood strategies. According to our data, the proportion of households adopting the livelihood strategy of livestock breeding was only 28.61%, which was a little higher than those adopting livestock breeding and off-farm activities. The third most popular livelihood strategy was the joint livelihood strategy of livestock breeding and crop planting and off-farm activities at a proportion of 24.94%. The least common livelihood strategy adopted by the households was livestock breeding and crop planting, at 20.05%. These findings show that livestock breeding is prevalent among the households around MMNR, which is consistent with previous studies [23,47,49]. However, the households around this reserve are more likely to adopt joint livelihood strategies rather than a single strategy. 

The adoption of a livelihood strategy seems to be related to the education level of the household head. About 30.2% of the households with an illiterate household head adopted the single strategy of livestock breeding, which was 3.67, 6.93, and 10.2 percentage points higher than those adopting a joint strategy of livestock breeding and off-farm activities, livestock breeding and crop planting and off-farm activities, and livestock breeding and crop planting, respectively. On the other hand, households with literate heads were more likely to choose diversified livelihood activities than those with illiterate heads. It can also be seen that households with higher education levels were more inclined to adopt a strategy involving off-farm activities (Table 2). 

The proportion of strategies involving off-farm activities for households living a considerable distance from the PA was higher than those adjacent to the PA. However, the difference is not significant. It is notable that households living adjacent to the PA preferred to adopt a joint strategy of breeding and crop planting with a proportion of 26.34%, which is higher than the proportion of 12.43% among those far away from the PA. This is probably due to the strict restrictions in the PA, resulting in fewer opportunities for off-farm activities. Households living distant to the PA were more inclined to choose livestock breeding as their single livelihood strategy, which may be due to the larger land areas that they own providing a natural basis for breeding.

The adoption of a livelihood strategy is also related to the household dependency ratio. This study used the median of household dependency ratio to group samples. It can be seen that the proportion of agricultural strategies selected by the low dependency ratio group was also lower. A total of 15.27% of households in the low dependency ratio group chose the joint strategy of livestock breeding and crop planting, which was 9.49% less than that of the high dependency ratio group. In addition, households with a low dependency ratio seemed to be more inclined to adopt livelihood strategies involving off-farm activities. The proportion of households adopting the joint strategy of livestock breeding and off-farm activities as well as the joint strategy of livestock breeding, crop planting, and off-farm activities was higher than that of the high dependency ratio group.

There are significant differences in per capita income among the households adopting different livelihood strategies. Specifically, the per capita income among the households adopting the joint strategy of livestock breeding and off-farm activities, livestock breeding and crop planting and off-farm activities, and livestock breeding and crop planting were 99,119 KES, 88,972 KES, and 86,318 KES, respectively, which was significantly larger than the 51,618 KES among the households adopting the single strategy of livestock breeding (Table 3). This finding implies that livelihood diversification is able to improve the income level of households around the PA, and the strategies with off-farm activities provide more benefit to household income. Additionally, it can also be seen that no matter what strategy was adopted, the income level of residents around the PA was far lower than the national average level of 168,921 KES [66], and there was a wide and deep level of poverty.

When adopting livelihood strategies involving off-farm activities, the households with literate household heads had a higher per capital income than those with illiterate heads among those adopting strategies that included off-farm activities. For example, the per capita income for households with literate heads adopting the strategy of livestock breeding and off-farm activities was up to 119,511 KES, nearly 40% higher than those with an illiterate head and the same livelihood strategy. As for the households that did not participate in off-farm activities, it is interesting that households with illiterate heads had higher incomes, which may be due to the fact that they spent more time breeding and planting compared with those receiving education and thus obtained improved agricultural output. 

No matter which strategy was adopted by the household, the income of households adjacent to the PA was higher than those distant to the PA. Especially for those adopting livestock breeding and off-farm activities, the average per capita income of the households adjacent to the PA was 116,740 KES, which was nearly 1.5 times of those distant to the PA. With the development of tourism around the PA, households in neighboring communities had access to more off-farm opportunities and gained higher income [23,64].

Similarly, for all types of strategies, low-dependency households tended to have higher income levels than high-dependency households, with significant differences. In particular, for the low dependency ratio group, the average per capita income from adopting the strategy of livestock breeding was 62,464 KES, almost twice as much as the high dependency ratio group. The large number of elderly individuals and children in the family could have a negative impact on household income [14].

### 4.2. Results of Empirical Models

#### 4.2.1. Determinants of Different Livelihood Strategies

According to the regression results, the direction and magnitude of the coefficient of determinants varied for different livelihood strategies (Table 4). 

Physical capital, natural capital, and financial capital had significant associations with the probability of adopting the strategy of livestock breeding. Specifically, the likelihood of adopting the strategy of livestock breeding decreased by 1 percentage point if the vehicle value increased by 1% (*p* < 0.01). This is probably because households that own more vehicles had the convenience to go out and get more means of livelihood. Similarly, the probability of adopting the strategy of livestock breeding decreased by 18.2 percentage points with a 1% addition in the value of agricultural production tools (*p* < 0.01). The agricultural production tool in this study included solar lighting equipment, spray pumps for livestock and crops, rainwater tanks, and hand carts. As for natural capital, the farmland area had a positive association with the probability of adopting the strategy of livestock breeding. The coefficient was 0.001, indicating a 0.1 percentage point increase in the probability of adopting the strategy of livestock breeding with a farmland area increase of 1 acre (*p* < 0.1). Whether a household has savings in banks or loans had a negative association with the probability of adopting the strategy of livestock breeding (13 percentage points). The households in all the other five wards were much more inclined to adopt the strategy of livestock breeding compared to those who lived in Angata.

All five types of livelihood capital were associated with the probability of adopting the strategy of livestock breeding and crop planting except for natural capital. For human capital, the higher the dependency ratio, the higher the probability of adopting this strategy (*p* < 0.05). A high dependency ratio reflects households with few laborers supporting many children and elders; these were more likely to choose livelihood strategies in order to take care of them [14]. Social capital had a significant association with the probability of adopting the strategy of livestock breeding and crops. The likelihood of adopting this strategy decreased by 0.5 percentage points with a one percent increase in the socializing expenditure (*p* < 0.1). Better interpersonal relationships may enable households to have more livelihood opportunities, for example, by bringing off-farm activities into livelihood strategies [67]. Physical capital had a significant and positive association with the probability of adopting this strategy (*p* < 0.01). For financial capital, if the household had savings in banks or loans, the probability of taking this strategy declined by 10.6 percentage points (*p* < 0.05). The households owning financial capital preferred not to engage in activities involving livestock breeding and crop planting, which may be due to the fact that financial capital gave them more possibilities of undertaking off-farm activities. In addition, the distance to the PA boundary had a negative effect on the probability of adopting this livelihood strategy. It decreased by 0.5 percentage points with a one-kilometer increase in the distance (*p* < 0.05). Contrary to the adoption of the strategy of livestock breeding, the households around all the other five wards preferred not to take this strategy compared to those located in Angata.

The probability of adopting the joint strategy of livestock breeding and off-farm activities was associated with human capital, social capital, physical capital, and financial capital. For human capital, the age of the household head had a negative association with the probability of adopting this strategy (*p* < 0.05). It seems that the elder household heads tended not to choose the joint strategy of livestock breeding and off-farm activities. When the expenditure on social activities increased by one percent, the probability of adopting this strategy increased by 0.9 percentage points (*p* < 0.01). For physical capital, contrary to adopting the strategy of livestock breeding, both vehicle value and agricultural production tool value had a significant and positive association with the probability of adopting this strategy. Specifically, if the vehicle value increased by one percent, the probability of adopting this strategy rose by 1.1 percentage points (*p* < 0.01). Similarly, it increased by 7.8 percentage points when the agricultural production tool value increased by one percent (*p* < 0.01). This finding may be due to the fact that the increase in modern equipment was conducive to reducing labor input, which increased off-farm activities [68]. Whether the household had savings in banks or loans had a positive association with the probability of adopting this strategy (*p* < 0.01). The households located in Angata had a lower probability of adopting this strategy than those located in the other five wards. 

The probability of adopting the joint strategy of livestock breeding, crop planting, and off-farm activities was associated with all five kinds of livelihood capital except for financial capital. For human capital, the older the household head, the greater the probability of taking this strategy (*p* < 0.01). Older people have much experience and may prefer to choose a richer portfolio of livelihood activities to avoid risks or create benefits. The education level of the household head had a negative association with the probability of adopting this strategy. It decreased by 8.2 percentage points if the household head was illiterate (*p* < 0.1). As for social capital, contributions to social activities had a negative association with the probability of adopting this strategy (*p* < 0.01). In addition, the likelihood of adopting this strategy increased by 0.7 percentage points with a one percent increase in the socializing expenditure (*p* < 0.05). This finding further validates that close contact with relatives and friends contributes to increased livelihood opportunities, especially off-farm activities. For physical capital, the probability of choosing this strategy increased by 6.3 percentage points if the agricultural production tool value increased by one percent (*p* < 0.01). As for natural capital, quite opposite to adopting the strategy of livestock breeding, farmland area had a significant and negative association with the probability of adopting this strategy (*p* < 0.05). Households that own more farmland tend to choose livelihood activities that are highly dependent on farmland, such as crop planting or breeding, to make a living rather than engage in off-farm employment [58,59]. Living farther away from the PA had a positive association with the probability of adopting this strategy (*p* < 0.01), which is probably due to the fact that off-farm activities are limited for households adjacent to the PA [69]. The households located in Angata had a higher probability of adopting this strategy than those located in the other five wards.

#### 4.2.2. The Association between Livelihood Strategies and Household Income and Its Heterogeneities

##### The Association between Livelihood Strategies and Household Income

According to the results of the OLS estimation, both the strategy of livestock breeding and off-farm activities and the strategy of livestock breeding, crop planting, and off-farm activities had a significant and positive association with the per capita household income around PAs (Table 5). The per capita income of households adopting the strategy of livestock breeding and off-farm activities, on average, was 58.3% higher than that of the households adopting the strategy of livestock breeding (*p* < 0.01). Similarly, households utilizing the strategy of livestock breeding, crop planting, and off-farm activities had a 42.3% higher income than those using the strategy of livestock breeding (*p* < 0.01). This indicates that participating in the joint strategy of livestock breeding and off-farm activities resulted in a greater improvement in household income.

Several variables of livelihood capital were correlated with per capita household income. The low education level of the household head was negatively related to household income. The total income decreased by 18.9% if the household head was illiterate (*p* < 0.1), since education plays a vital role in increasing household income [60,61,62]. The dependency ratio also had a negative effect on the household income. Per capita income decreased by 0.8% with a one percentage point increase in the dependency ratio (*p* < 0.01). Physical capital had a significant and positive association with the total income of a household. Households with high vehicle values were more likely to have a high household income (*p* < 0.1). As for distance to the PA, the closer the households living to the PA, the higher the per capita income. Specifically, the per capita income of households decreased by 0.9% if the distance to PA increased by one km (*p* < 0.1). It is important to note that although the protection constraints of the PA reduced the off-farm opportunities available to nearby residents, tourism was developing well in areas close to the PA; in contrast, for the areas far away from the PA, the tourism industry was less developed and the off-farm opportunities relying on tourism were limited [18,64]. The households around Naikarra and Siana had 41.4 and 53.6% lower per capital household income than those in Angata, respectively (*p* < 0.05).

##### The Heterogeneity of the Association between Livelihood Strategies and Household Income

According to the above analysis, both the strategy of livestock breeding and off-farm activities and the strategy of livestock breeding, crop planting, and off-farm activities had positive associations with per capita household income. Did these two strategies have the same association with the income of the households living adjacent to the PA and those living far from the PA? How did strategies affect total income for households with an illiterate or literate household head? Was there any difference in the impact of strategy adoption on income for households with different dependency ratios? To answer these questions, Table 6 showed the heterogeneities by the education level of the household head, the distance to the PA, and the dependency ratio of the associations between livelihood strategies and per capital household income. In view of the generally low education level in the study area, the education level of the household head was simply divided into the two groups of illiterate and literate. 

The results of the OLS estimation show that adopting the strategy of livestock breeding and off-farm activities and the strategy of livestock breeding, crop planting, and off-farm activities had different associations with income among the households with different education levels of household heads, different distances to the PA, and different dependency ratios. Specifically, compared to the adoption of the strategy of livestock breeding, adopting the joint strategy of livestock breeding and off-farm activities was able to increase per capita income by 38.1% for the households with an illiterate household head (*p* < 0.05) and by 80% for those with a literate household head (*p* < 0.01). This indicates that adopting this strategy had a much greater association with income for households with a literate household head. The heterogeneity of adopting the strategy of livestock breeding, crop planting, and off-farm activities also showed a similar pattern as the strategy of livestock breeding and off-farm activities. Under this strategy, the per capita income rose by 41.4% if the household head was illiterate (*p* < 0.05), and it increased by 55.7% when the household head was literate (*p* < 0.05).

With regard to the distance to the PA, it can be seen that both of these two strategies had a positive association with the per capital household income for adjacent and distant households. When the strategy of livestock breeding was set as the reference group, adopting the strategy of livestock breeding and off-farm activities increased the household income by 68% (*p* < 0.01) and 44% (*p* < 0.05) for adjacent households and distant households, respectively. The income of households living far from the PA increased less than the adjacent households because there were less tourism and fewer opportunities for off-farm activities in areas far away from the PA. As for the strategy of livestock breeding, crop planting, and off-farm activities, it was able to increase household income by 37.3% (*p* < 0.1) for adjacent households and 39.6% (*p* < 0.01) for distant households when compared to the strategy of livestock breeding. 

As for the dependency ratio, similar to distance to the PA, both strategies had a positive impact on per capita household income for households regardless of low or high dependency ratio. Compared to the strategy of livestock breeding, adopting the strategy of livestock breeding and off-farm activities increased household income by 57% (*p* < 0.01) and 65.3% (*p* < 0.01) for low-dependency ratio households and high-dependency ratio households, respectively. Livestock breeding requires less physical strength, so the elderly and children are more likely to participate in this activity, which may be the main reason for this result. When adopting the strategy of livestock breeding, crop planting, and off-farm activities, it can be seen that the low-dependency ratio group could increase the household income by 47.7% (*p* < 0.05), which was greater than 40.2% (*p* < 0.05) of the high-dependency ratio group.

In this article, the interaction terms of distance and strategy, education level and strategy, and dependency ratio and strategy were added to the regression models. However, the coefficients were not significant (Table A1), which means that the strategies involving off-farm activities were inclusive.

## 5. Discussion

The results from the Mlogit regressions showed that the determinants of the four strategies varied. The age of the household head had a significant and negative association with the probability of adopting the strategy of livestock breeding and off-farm activities, while it had a positive association with the probability of adopting the strategy of livestock breeding, crop planting, and off-farm activities. There are three possible reasons to explain this finding. First, the strategy of livestock breeding, crop planting, and off-farm activities increased crop planting compared with the strategy of livestock breeding and off-farm activities. Livestock breeding consumes more energy compared with crop planting and also requires less physical strength and is much more suitable for the elderly. Second, older household heads were more experienced, hence preferring to choose more diversified livelihood activities in order to avoid risk or increase income sources. Third, older people usually have affection for farmland and view the land as crucial for survival, so they often retain the activity of crop planting even if there are other livelihood activities available to them [70]. 

Contribution to social activities played an important role in increasing the likelihood of adopting the strategy of livestock breeding and off-farm activities but decreased the probability of adopting the strategy of livestock breeding, crop planting, and off-farm activities. This is probably because affiliating with a certain group allows households to easily obtain more external information, thus improving the possibility of off-farm employment [10,57]. On the other hand, households participating in the same religion had strong homogeneity and tended to adopt a specific livelihood strategy other than attempt diversification [71]. 

As for the effects of physical capital, vehicle value had a negative association with the probability of adopting the strategy of livestock breeding, while a positive association with the probability of adopting the strategy of livestock breeding and off-farm activities. This is mainly caused by the fact that more vehicles can aid people in accessing more off-farm work. In addition, agricultural production tool value decreased the probability of adopting a strategy of livestock breeding, which implies that rich agricultural production tools can support residents in conducting diversified activities.

Financial capital also played a crucial role in strategy decisions. If a household had savings in banks or loans, the probability of adopting the strategy of livestock breeding and off-farm activities increased, while the probability of adopting a strategy only related to agricultural activities decreased. It is obvious that owning financial capital enabled households to increase off-farm activities [57].

Living farther away from the PA had a negative association with the probability of adopting the strategy of livestock breeding and crop planting, but a positive association with the probability of adopting the strategy of livestock breeding, crop planting, and off-farm activities. This may be because the further away from the PA, the fewer constraints the households were subject to and the more opportunities they had to engage in other activities other than breeding and planting [8,14], resulting in the high probability of choosing a strategy involving off-farm activities.

The results from OLS regression showed that compared with the single livelihood activity of livestock breeding, diversified livelihood strategies, especially strategies involving off-farm activities, had a more significant effect on increasing household income. However, the high diversification of livelihood does not always mean high income, and it can be seen that the joint strategy of livestock breeding and off-farm activities had a greater impact on household income than the joint strategy of livestock breeding, crop planting, and off-farm activities, indicating that regarding types of livelihood strategies, it is not necessarily the more the better but also depends on the actual situation of the region.

Furthermore, when considering the heterogeneity of the impact of livelihood strategies on household income, adopting the strategy of livestock breeding and off-farm activities had a much greater association with income for the households with a literate household head. This further demonstrates the importance of education in increasing household income by diversifying livelihood strategy. 

The findings of this study provide several practical implications. Livelihood diversification improves the household income around the MMNR, especially with strategies involving off-farm activities. It is thus necessary to encourage the local residents to increase the types of livelihood activities they adopt. The government and management authority of Maasai Mara National Reserve should provide households around the PA with more off-farm employment opportunities, such as ecotourism, especially in areas far away from the PA, to increase the welfare of residents as well as to utilize natural resources sustainably. Furthermore, policies improving the educational level of household heads should be implemented to reduce poverty and enhance capacity for income generation. Moreover, local governments should improve public transportation to help the residents undertake more livelihood activities. This article also adds theoretical implications to the existing literature. Previous studies on the determinants of livelihood outcomes generally only discussed livelihood capital when performing empirical research. However, in this study, livelihood strategy was added to the analysis, and it can be verified that livelihood strategy also has a significant impact on livelihood outcomes, which further enriches the research framework in the literature.

## 6. Conclusions

In this study, the determinants of four livelihood strategies were analyzed and the association between each type of strategy and household income was estimated by adopting the use of Mlogit regression and OLS estimation based on the data collected from 409 households in six wards around the Maasai Mara National Reserve. 

It can be seen that the determinants of the four strategies varied, reflected by the directions and magnitudes of the effects of the five types of livelihood capital on different strategies. There were significant differences in the strategies that involved off-farm activities and those that did not. Diversification strategies, especially those involving off-farm activities, played a greater role in raising household income. Furthermore, these strategies had better associations with income for the households with literate household heads and those adjacent to the PA. 

We acknowledge that there are still some limitations to this study. First, there is a lack of basic information concerning the six wards in the survey, thus omitting to consider the influence of ward characteristics, such as development level. Second, the effects of diversification on different income categories, such as agricultural income and off-farm income, remain unexplored. These factors should be further analyzed in the future to provide more targeted implications for management authorities. Third, only one year’s worth of data was investigated in this study. Investigating diversified livelihood choices and the impact on household welfare after the COVID-19 pandemic should be a priority in future research.

## Figures and Tables

**Table 1:** Descriptive statistics.

Variable	Definition	Mean	Std. Dev.	Min.	Max.
Strategy					
Strategy2	Livestock breeding and Crop planting	0.20	0.40	0	1
Strategy3	Livestock breeding and Off-farm activities	0.26	0.44	0	1
Strategy4	Livestock breeding and Crop planting and Off-farm activities	0.25	0.43	0	1
Income					
Totalincome	Household income (KES)	427,963	407,988	12,000	3,012,300
Perincome	Per capita household income (KES)	80,434	85,524	2286	641,000
lnperincome	Log of per capita household income	10.85	1.00	7.73	13.37
Human capital					
Age	Age of household head	43.64	13.28	20	95
Illiterate	Whether the household head is illiterate (1 = yes; 0 = no)	0.60	0.49	0	1
Perfeed1	Household dependency ratio (percent)	38.80	22.46	0	100
Social capital					
lnsocialcontribution	Log of contribution to social activities (membership fee to community groups such as church and chama)	2.66	7.03	−4.61	12.43
lnsocializing	Log of socializing (entertainment of friends and family)	2.89	6.81	−4.61	12.10
Physical capital					
lntrans	Log of vehicle value	2.97	8.07	−4.61	14.89
lnproduct	Log of agricultural production tool value	−4.00	3.04	−4.61	14.73
Natural capital					
Farmland	Farmland area (acres)	66.06	67.78	0	700
Financial capital					
loansaving	Whether the household have savings in banks or loans associations? (1 = yes; 0 = no)	0.22	0.41	0	1
dist	Distance of the interviewee’s house to the PA boundary (km)	8.94	8.55	0	25
dist1	Whether the household is adjacent to the PA? (1 = yes; 0 = no)	0.55	0.50	0	1
Wards					
Kimintet	Whether the household is located in this ward (1 = yes; 0 = no)	0.12	0.33	0	1
Lolgorian	Whether the household is located in this ward (1 = yes; 0 = no)	0.33	0.47	0	1
Mara	Whether the household is located in this ward (1 = yes; 0 = no)	0.11	0.32	0	1
Naikarra	Whether the household is located in this ward (1 = yes; 0 = no)	0.15	0.36	0	1
Siana	Whether the household is located in this ward (1 = yes; 0 = no)	0.14	0.35	0	1

**Table 2:** Proportion of households around MMNR adopting different livelihood strategies (%).

Strategy	Livestock Breeding	Livestock Breeding and Crop Planting	Livestock Breeding and Off-Farm Activities	Livestock Breeding and Crop Planting and Off-Farm Activities	*p* Value
Total	28.61	20.05	26.41	24.94	
Education					
Illiterate	30.2	20	26.53	23.27	0.3009
Literate	26.22	20.12	26.22	27.44	
Distance to the PA					
Adjacent	25.45	26.34	25.89	22.32	0.6173
Distant	32.43	12.43	27.03	28.11	
Dependency ratio					
Low	31.03	15.27	27.59	26.11	0.8493
High	26.21	24.76	25.24	23.79	

**Table 3:** Income of households adopting different livelihood strategies (KES).

Income	Livestock Breeding	Livestock Breeding and Crop Planting	Livestock Breeding and Off-Farm Activities	Livestock Breeding and Crop Planting and Off-Farm Activities	*p* Value
Total	51,618	86,318	99,119	88,972	
Education					
Illiterate	54,147	87,749	85,629	83,916	0.2156
Literate	47,266	84,193	119,511	95,377	
Distance					
Adjacent	55,674	91,018	116,740	104,535	0.0033
Distant	47,764	74,260	78,679	74,008	
Dependency ratio					
Low	62,464	90,505	109,427	92,474	0.0955
High	38,964	83,772	88,019	85,185	

**Table 4:** Marginal effects of different strategies.

Variables	Strategy 1	Strategy 2	Strategy 3	Strategy 4
Human capital				
Age	−0.002	0.001	−0.003 **	0.005 ***
	(−1.086)	(0.518)	(−2.053)	(2.905)
Illiterate	0.007	0.015	0.060	−0.082 *
	(0.149)	(0.382)	(1.424)	(−1.931)
Perfeed1	−0.001	0.002 **	0.000	−0.001
	(−0.717)	(2.034)	(0.184)	(−1.110)
Social capital				
lnsocialcontribution	0.002	−0.000	0.009 ***	−0.011 ***
	(0.616)	(−0.011)	(2.669)	(−3.576)
lnsocializing	−0.003	−0.005 *	0.001	0.007 **
	(−0.867)	(−1.751)	(0.327)	(2.211)
Physical capital				
lntrans	−0.010 ***	−0.001	0.011 ***	0.000
	(−4.202)	(−0.353)	(4.955)	(0.079)
lnproduct	−0.182 ***	0.041 ***	0.078 ***	0.063 ***
	(−15.899)	(5.682)	(8.516)	(7.954)
Natural capital				
Farmland	0.001 *	0.000	0.000	−0.001 **
	(1.868)	(0.748)	(0.463)	(−2.013)
Financial capital				
loansaving	−0.130 **	−0.106 **	0.175 ***	0.062
	(−2.405)	(−1.966)	(4.428)	(1.335)
dist	0.000	−0.005 **	−0.000	0.005 *
	(0.059)	(−1.971)	(−0.062)	(1.854)
Region				
Kimintet	0.316 ***	−0.408 ***	0.432 ***	−0.341 ***
	(5.092)	(−5.342)	(6.739)	(−4.618)
Lolgorian	0.238 ***	−0.209 ***	0.165 ***	−0.194 ***
	(5.458)	(−2.882)	(4.836)	(−2.657)
Mara	0.374 ***	−0.506 ***	0.535 ***	−0.404 ***
	(5.068)	(−7.917)	(7.193)	(−4.893)
Naikarra	0.329 ***	−0.448 ***	0.321 ***	−0.202 **
	(5.455)	(−6.107)	(6.822)	(−2.547)
Siana	0.421 ***	−0.467 ***	0.360 ***	−0.314 ***
	(6.250)	(−6.568)	(6.752)	(−3.726)
Observations	409	409	409	409

Note: *, **, and *** denote significance levels at 10, 5, and 1 percent.

**Table 5:** The impact of different livelihood strategies on total household income.

	Dependent Variable: Log of per Capita Household Income			
Variables	Model 1	Model 2	Model 3	Model 4
Strategy				
Strategy 2	0.454 ***	0.467 ***	0.407 ***	0.222
	(2.925)	(3.026)	(2.619)	(1.288)
Strategy 3	0.713 ***	0.554 ***	0.564 ***	0.583 ***
	(5.733)	(4.189)	(4.336)	(4.560)
Strategy 4	0.618 ***	0.524 ***	0.521 ***	0.423 ***
	(4.906)	(3.997)	(3.993)	(3.147)
Human capital				
Age		0.002	0.001	0.001
		(0.319)	(0.151)	(0.229)
Illiterate		−0.181 *	−0.185 *	−0.189 *
		(−1.691)	(−1.751)	(−1.864)
Perfeed1		−0.008 ***	−0.009 ***	−0.008 ***
		(−3.040)	(−3.159)	(−3.015)
Social capital				
lnsocialcontribution		0.015	0.011	0.006
		(1.642)	(1.242)	(0.664)
lnsocializing		0.001	0.003	0.007
		(0.085)	(0.336)	(0.801)
Physical capital				
lntrans		0.010	0.011 *	0.011 *
		(1.586)	(1.734)	(1.774)
lnproduct		0.019	0.020	0.016
		(1.132)	(1.267)	(1.026)
Natural capital				
Farmland		0.000	0.000	0.001
		(0.671)	(0.695)	(1.291)
Financial capital				
loansaving		0.138	0.154	0.117
		(1.250)	(1.422)	(1.107)
dist			−0.015 ***	−0.009 *
			(−3.034)	(−1.815)
Region				
Kimintet				−0.239
				(−1.155)
Lolgorian				0.140
				(1.041)
Mara				−0.004
				(−0.019)
Naikarra				−0.414 **
				(−2.565)
Siana				−0.536 ***
				(−3.172)
Constant	10.412 ***	10.786 ***	10.981 ***	11.039 ***
	(111.869)	(38.307)	(39.009)	(39.484)
	409	409	409	409
R-squared	0.083	0.151	0.167	0.219

Note: *, **, and *** denote significance levels at 10, 5, and 1 percent.

**Table 6:** Heterogeneities in the association between livelihood strategies and household income.

	Dependent Variable: Log of per Capita Household Income					
Variables	Illiterate	Literate	Adjacent	Distant	Low Dependency Ratio	High Dependency Ratio
Strategy						
Strategy 2	0.164	0.288	0.058	0.231	0.244	0.231
	(0.822)	(0.851)	(0.260)	(0.878)	(0.965)	(0.967)
Strategy 3	0.381 **	0.851 ***	0.680 ***	0.440 **	0.570 ***	0.653 ***
	(2.286)	(4.102)	(3.739)	(2.306)	(2.794)	(3.564)
Strategy 4	0.414 **	0.557 **	0.373 *	0.396 **	0.477 **	0.402 **
	(2.399)	(2.342)	(1.872)	(2.270)	(2.266)	(2.350)
Regional dummies	Yes	Yes	Yes	Yes	Yes	Yes
Control variables	Yes	Yes	Yes	Yes	Yes	Yes
Constant	10.754 ***	11.237 ***	10.788 ***	12.044 ***	10.944 ***	10.435 ***
	(28.298)	(25.039)	(31.227)	(33.624)	(33.594)	(29.320)
Observations	245	164	224	185	203	206
R-squared	0.221	0.283	0.230	0.363	0.180	0.252

Note: *, ** and *** denote significance levels at 10, 5, and 1 percent. The regional dummies and control variables are the same as those in Table 4.

## Data Availability

Data may be made available from the corresponding author upon reasonable request by researchers.

## Appendix A

**Table A1:** Heterogeneity results.

Dependent Variable: Log of per Capita Household Income			
Variables	Illiterate/Literate	Adjacent/Distant	Low/High Dependency ratio
Strategy			
Strategy 2	0.325	0.286	−0.132
	(1.136)	(1.176)	(−0.397)
Strategy 3	0.801 ***	0.444 **	0.467 *
	(3.975)	(2.558)	(1.949)
Strategy 4	0.486 **	0.308 *	0.228
	(2.365)	(1.771)	(0.956)
Illiterate	−0.034		
	(−0.183)		
Strategy 2*illiterate	−0.164		
	(−0.500)		
Strategy 3*illiterate	−0.362		
	(−1.479)		
Strategy 4*illiterate	−0.092		
	(−0.376)		
dist1		0.037	
		(0.209)	
Strategy 2*dist1		−0.079	
		(−0.260)	
Strategy 3*dist1		0.250	
		(1.059)	
Strategy 4*dist1		0.218	
		(0.894)	
Perfeed 1			−0.012 ***
			(−3.192)
Strategy 2*perfeed			0.009
			(1.250)
Strategy3* perfeed			0.003
			(0.623)
Strategy 4*perfeed			0.005
			(0.963)
Reginal dummies	Yes	Yes	Yes
Control variables	Yes	Yes	Yes
Constant	10.937 ***	10.952 ***	11.100 ***
	(34.794)	(36.713)	(38.832)
Observations	409	409	409
R-squared	0.223	0.223	0.219

Note: *, **, and *** denote significance levels at 10, 5, and 1 percent.
